# ​Fusarium Protein Toolkit: a web-based resource for structural and variant analysis of *Fusarium* species

**DOI:** 10.1186/s12866-024-03480-5

**Published:** 2024-09-06

**Authors:** Hye-Seon Kim, Olivia C. Haley, John L. Portwood II, Stephen Harding, Robert H. Proctor, Margaret R. Woodhouse, Taner Z. Sen, Carson M. Andorf

**Affiliations:** 1 USDA, Agricultural Research Service, National Center for Agricultural Utilization Research, Mycotoxin Prevention and Applied Microbiology Research Unit, 1815 N University St, Peoria, IL 61604 USA; 2 USDA, Agricultural Research Service, Corn Insects and Crop Genetics Research Unit, 819 Wallace Rd. Ames, IA, 50011 USA; 3 USDA, Agricultural Research Service, Crop Improvement and Genetics Research Unit, 800 Buchanan St. Albany, CA, 94710 USA; 4 Department of Bioengineering, University of California, 306 Stanley Hall, Berkeley, CA 94720 USA; 5 Department of Computer Science, Iowa State University, 2434 Osborn Dr, Ames,, IA, 50011 USA

**Keywords:** Fusarium, Proteomics, Protein structures, Variant effects, Pan-genome

## Abstract

**Background:**

​​The genus *Fusarium* poses significant threats to food security and safety worldwide because numerous species of the fungus cause destructive diseases and/or mycotoxin contamination in crops. The adverse effects of climate change are exacerbating some existing threats and causing new problems. These challenges highlight the need for innovative solutions, including the development of advanced tools to identify targets for control strategies.

**Description:**

In response to these challenges, we developed the Fusarium Protein Toolkit (FPT), a web-based tool that allows users to interrogate the structural and variant landscape within the *Fusarium* pan-genome. The tool displays both AlphaFold and ESMFold-generated protein structure models from six *Fusarium* species. The structures are accessible through a user-friendly web portal and facilitate comparative analysis, functional annotation inference, and identification of related protein structures. Using a protein language model, FPT predicts the impact of over 270 million coding variants in two of the most agriculturally important species, *Fusarium graminearum* and *F. verticillioides*. To facilitate the assessment of naturally occurring genetic variation, FPT provides variant effect scores for proteins in a *Fusarium* pan-genome based on 22 diverse species. The scores indicate potential functional consequences of amino acid substitutions and are displayed as intuitive heatmaps using the PanEffect framework.

**Conclusion:**

FPT fills a knowledge gap by providing previously unavailable tools to assess structural and missense variation in proteins produced by *Fusarium*. FPT has the potential to deepen our understanding of pathogenic mechanisms in *Fusarium*, and aid the identification of genetic targets for control strategies that reduce crop diseases and mycotoxin contamination. Such targets are vital to solving the agricultural problems incited by *Fusarium*, particularly evolving threats resulting from climate change. Thus, FPT has the potential to contribute to improving food security and safety worldwide.

**Supplementary Information:**

The online version contains supplementary material available at 10.1186/s12866-024-03480-5.

## Background

The genus *Fusarium* includes some of the fungi of most concern to agriculture because of their ability to cause crop diseases and contaminate crops with mycotoxins that are health hazards to humans and livestock. Most crops have at least one economically important disease caused by a *Fusarium* species [1]. One of the most important species, *F. graminearum*, causes Fusarium head blight (FHB) of cereal crops, which reduces yield and contaminates grain with the mycotoxin deoxynivalenol. Losses caused by this fungus vary yearly, but severe FHB epidemics in the US were estimated to cause losses of almost $3 billion in the 1990s [2] and $1.5 billion in 2015–2016 [3]. In Europe, deoxynivalenol contamination alone caused an estimated loss of €3 billion to wheat production [4]. Another species, *F. verticillioides*, causes Fusarium ear rot of maize and contaminates kernels with fumonisin mycotoxins. Economic losses caused by the latter species and fumonisin contamination are not as well documented as those caused by FHB, but losses were estimated to be as high as $135 million in the High Plains of Texas in 2017 [5].

Climate change is expected to increase the susceptibility of crops to *Fusarium*-incited diseases and mycotoxin contamination, thereby amplifying their negative economic impact [6]. This is because climate change alters temperature and precipitation patterns, creating favorable conditions for *Fusarium* growth and reproduction, and increasing the likelihood of pathogen-crop interactions. Additionally, climate-related stress can weaken crop immune systems [7], making them more vulnerable to *Fusarium* infection. An integrated approach that leverages scientific knowledge and technological innovation is essential to address the negative impacts of *Fusarium* on agriculture. Understanding the dynamics of the interactions of *Fusarium* species and their crop hosts on a molecular level is critical for developing effective control strategies to mitigate the adverse economic and health impacts caused by these fungi.

A significant knowledge gap for *Fusarium* is the absence of a comprehensive resource for searching and visualizing protein structures and exploring the functional consequences of amino acid substitutions within these proteins. This gap limits understanding of pathogenesis-related proteins in *Fusarium*, which in turn hinders the development of control strategies based on molecular mechanisms of pathogenesis. Recent advances, notably AlphaFold [8] and other technologies, have facilitated accurate predictions of protein structures, broadening the possibilities for comparative structural studies of proteins. The AlphaFold Protein Structure Database [9] now offers protein structures for over 200 million proteins from many organisms. New tools like the novel structural alphabet and tertiary interaction-based FoldSeek [10], offer protein alignment capabilities that are faster than previous methods.

Extending these tools to predict functional effects of missense variants —i.e., codon variations that cause amino acid substitutions — is crucial for understanding the molecular mechanisms of *Fusarium* evolution, pathogenesis, and adaptation, and could potentially reveal new targets for disease management. Missense variant approaches have been explored in research on human biology [11–13] but not *Fusarium* biology. The AlphaMissense [11] resource leverages structural and evolutionary information to classify all possible missense variants in the human genome as either benign or deleterious. The GEMME [12] and the esm-variants [13] workflows are other examples of alignment-based strategies and large-scale protein language models that predict mutational outcomes across numerous protein families and the entire human proteome, respectively. However, a tool that facilitates exploration of publicly available *Fusarium* proteomes and predicts potential functional consequences of missense changes in codons is yet to be developed.

A new resource tailored for *Fusarium* research should incorporate innovative technologies that enable the exploration of *Fusarium* protein structures and missense variants. Such a resource would not only facilitate the identification and visualization of protein structures but also enable predictions of the functional implications of genetic variants. By combining structural predictions with variant effect prediction technologies, this tool would likely enhance our understanding of molecular mechanisms of *Fusarium* pathogenicity, which could lead to the identification of targets for control. Such a resource would be an advancement in *Fusarium* research, offering insights into the genetic basis of variation in these pathogens and potentially informing efforts to develop innovative control measures.

Here, we introduce a new database resource called the Fusarium Protein Toolkit (FPT - https://fusarium.maizegdb.org/*)* which offers a suite of tools for exploring protein structures, variant effects, and annotated effector proteins from the genus *Fusarium*. FPT leverages the frameworks and tools developed for maize (*Zea mays*) [14, 15] by the Maize Genetics and Genomics Database (MaizeGDB) [16–19]. This shared infrastructure sets a foundation for future studies focused on *Fusarium*-maize interactions and can be effortlessly implemented in other biological databases such as GrainGenes [20]. FPT can be used as a stand-alone resource to explore *Fusarium* proteins or to facilitate comparisons of protein structures and functionalities between *Fusarium* and maize. These comparisons have the potential to provide an in-depth understanding of the molecular interactions between these organisms.

### Construction and content

#### Data sources and curation

The main features of the Fusarium Protein Toolkit (FPT) include predicted three-dimensional (3D) structures of proteins, which aid determination of function, and predictions of whether amino acid substitutions in orthologous proteins impact protein function. For the development of FPT, the following primary data types were collected or generated, and curated for *Fusarium* proteomes: (1) Effector proteins, (2) protein structure models, (3) pan-genome framework with protein alignments, orthology groups, and variant effect scores. Table 1 provides an overview of the 22 *Fusarium* species integrated into the FPT and Table 2 lists the data and tools available for each species. Supplementary Figure S1 lists which datasets were specifically curated for the FPT and which were acquired externally, such as the sequence downloads from GenBank or UniProt and the AlphaFold structure downloads from the AlphaFold Protein Structure Database.

**Table 1:** Overview of *Fusarium* Species in the Fusarium Protein Toolkit

Species	Taxonomy	UniProt	Protein Count
*Fusarium acutatum*	78,861	UP000536711	14,072
*Fusarium avenaceum*	40,199	UP000782241	11,232
*Fusarium coffeatum*	231,269	UP000253153	11,778
*Fusarium culmorum*	5516	UP000241587	12,350
*Fusarium duplospermum*	1,325,734	UP000288168	16,262
*Fusarium flagelliforme*	2,675,880	UP000265631	13,039
*Fusarium fujikuroi*	1,279,085	UP000016800	14,792
*Fusarium graminearum*	229,533	UP000070720	16,422
*Fusarium mangiferae*	192,010	UP000184255	15,798
*Fusarium musae*	1,042,133	UP000827133	13,672
*Fusarium odoratissimum*	1,089,451	UP000030685	19,807
*Fusarium oxysporum*	426,428	UP000009097	23,111
*Fusarium poae*	36,050	UP000091967	14,048
*Fusarium proliferatum*	1,227,346	UP000183971	16,122
*Fusarium pseudograminearum*	1,028,729	UP000007978	12,448
*Fusarium redolens*	48,865	UP000720189	17,005
Fusarium vanettenii*	660,122	UP000005206	15,711
*Fusarium sporotrichioides*	5514	UP000266152	11,960
*Fusarium subglutinans*	42,677	UP000547976	14,039
*Fusarium tjaetaba*	1,567,544	UP000530670	14,180
*Fusarium venenatum*	56,646	UP000245910	13,945
*Fusarium verticillioides*	334,819	UP000009096	17,876

This table provides a list of the 22 *Fusarium* species accessible via the Fusarium Protein Toolkit. It includes details for each species, such as the full species name (from UniProt), the NCBI Taxonomy Database ID, the UniProt Proteome ID, and the total number of proteins in each proteome from UniProt. *Formerly *F. solani f. sp. pisi* and *Nectria haematoccoca*

**Table 2:** Data availability for *Fusarium* Species in the Fusarium Protein Toolkit

Species	FASTA	PDB	Variant scores	Effectors	FoldSeek	PanEffect
*F. acutatum*	Y	N	Y	N	N	Y
*F. avenaceum*	Y	N	Y	N	N	Y
*F. coffeatum*	Y	N	Y	N	N	Y
*F. culmorum*	Y	N	Y	N	N	Y
*F. duplospermum*	Y	N	Y	N	N	Y
*F. flagelliforme*	Y	N	Y	N	N	Y
*F. fujikuroi*	Y	Y	Y	Y	Y	Y
*F. graminearum*	Y	Y	Y	Y	Y	Y
*F. mangiferae*	Y	N	Y	N	N	Y
*F. musae*	Y	N	Y	N	N	Y
*F. odoratissimum*	Y	N	Y	N	N	Y
*F. oxysporum*	Y	Y	Y	Y	Y	Y
*F. poae*	Y	N	Y	N	N	Y
*F. proliferatum*	Y	Y	Y	Y	Y	Y
*F. pseudograminearum*	Y	N	Y	N	N	Y
*F. redolens*	Y	N	Y	N	N	Y
F. vanettenii*	Y	Y	Y	Y	Y	Y
*F. sporotrichioides*	Y	N	Y	N	N	Y
*F. subglutinans*	Y	N	Y	N	N	Y
*F. tjaetaba*	Y	N	Y	N	N	Y
*F. venenatum*	Y	N	Y	N	N	Y
*F. verticillioides*	Y	Y	Y	Y	Y	Y

This table outlines the datasets available for download for the 22 *Fusarium* species featured in the Fusarium Protein Toolkit. It details the types of data accessible (including FASTA protein sequences, ESMFold protein structures in the PDB format, and variant effect scores) and specifies which species have been integrated into the Effector tables, FoldSeek search, and PanEffect tools. *Formerly *F. solani f. sp. pisi* and *Nectria haematoccoca*

#### Prediction of effector proteins

**Fig. 1:**
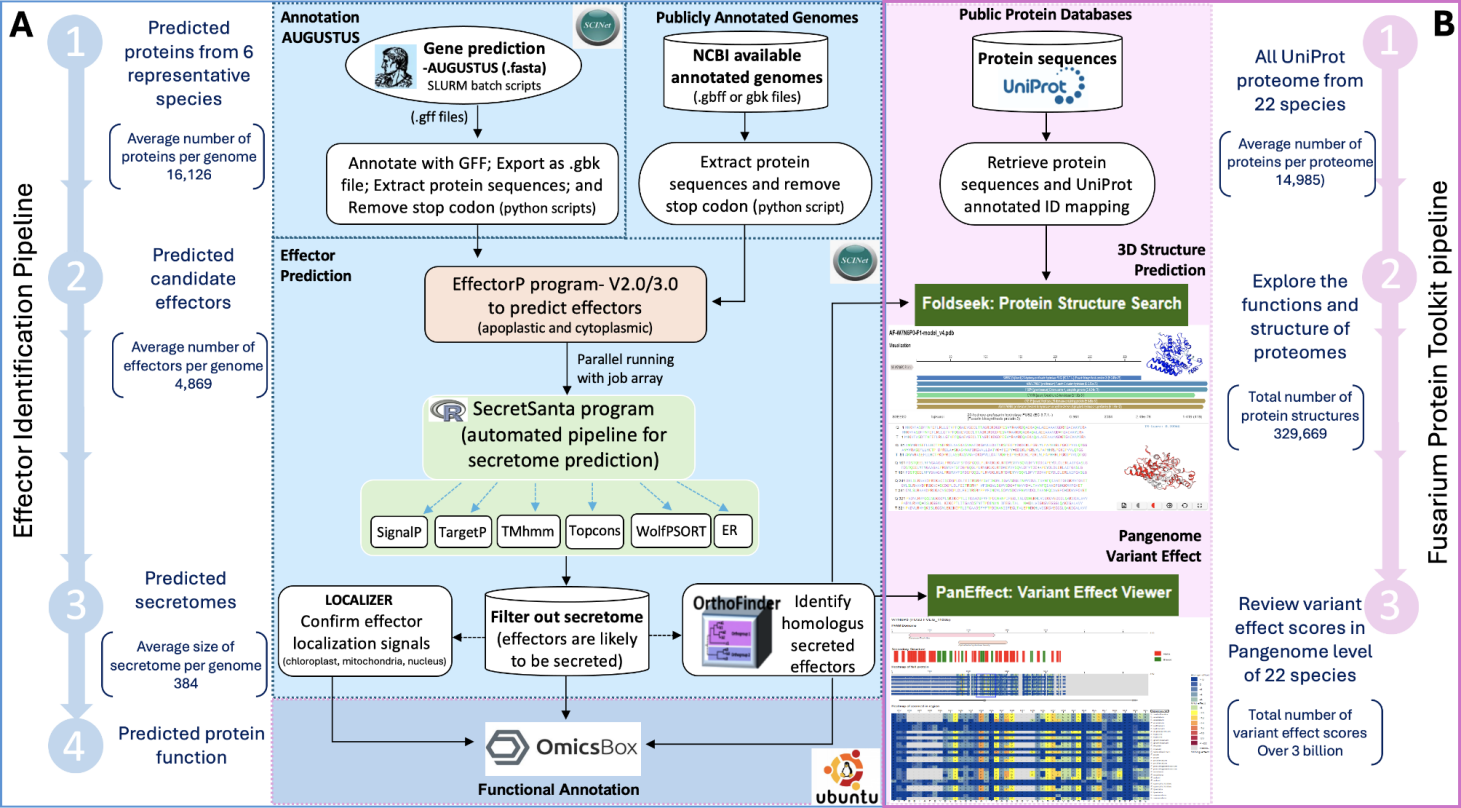
Pipeline for identification effectors and the Fusarium Protein Toolkit. (A) Effector Identification Pipeline: (1) Gene prediction with AUGUSTUS (2) Identification of candidate effectors with EffectorP (3) Identification of putative secretomes with SecretSanta, identification of effector orthologs with OrthoFinder and confirmation effector localization signals with LOCALIZER (4) Functional annotation with OmicsBox. (B) Fusarium Protein Toolkit: (1) Data retrieval from UniProt Protein database (2) Search function that enables the visualization of 3D protein structure of a protein of interest and structural alignment (3) Search function that provides potential impacts of missense variant effect scores in a genome and pan-genome level of 22 *Fusarium* species

Like other plant pathogenic fungi, *Fusarium* species secrete small proteins known as effectors that overcome plant defenses and, thereby, enable the fungi to cause disease. Genomic data from fungal plant pathogens has been used to identify effectors and aid efforts to understand the mechanism by which they impact plant defenses. However, genomic-based studies of *Fusarium* are limited and there are no genome-wide analyses of effectors from multiple species. Therefore, we used a combination of genome sequence analyses and machine learning approaches to develop a four-step computational pipeline to identify effectors, assess ortholog distribution, and predict potential function (Panel A of Fig. 1 shows this workflow). The genome sequences for six representative *Fusarium* species (*F. fujikuroi*, *F. graminearum*, *F. oxysporum*, *F. proliferatum*, *F. vanettenii*, and *F. verticillioides*) were downloaded from the NCBI Reference Sequence Database (RefSeq) [21]. Table 3 provides an overview of the predicted proteins, effector proteins, and secreted proteins in these species.

The four-step effector protein prediction pipeline is described below.

**Table 3:** Overview of the *Fusarium* species used in the effector annotation pipeline

Species	NCBI RefSeq	Total proteins	EffectorP proteins	SecretSanta proteins
*F. fujikuroi*	GCF_900079805.1	14,813	4,391	390
*F. graminearum*	GCF_000240135.3	13,313	4,424	290
*F. oxysporum*	GCF_000149955.1	20,925	6,799	462
*F. proliferatum*	GCF_900067095.1	16,125	4,711	439
F. vanettenii*	GCF_000151355.1	15,708	4,002	308
*F. verticillioides*	GCA_000149555.1	15,869	4,884	412

The table shows the six *Fusarium* species used to calculate putative effector proteins along with the NCBI RefSeq ID, the total number of proteins downloaded from RefSeq, the number of proteins annotated as likely effectors by EffectorP, and the number of effector proteins identified as likely secretors by SecretSanta. The proteins in the final column are used as the curated dataset in the Fusarium Protein Toolkit website. *Formerly *F. solani f. sp. pisi* and *Nectria haematoccoca*

- Step 1: **Predicted proteins from six representative species.** The genome annotations of the six representative *Fusarium* species are downloaded from NCBI (as GBFF or GBK files). The gene finder program AUGUSTUS [22] is used to identify additional gene models (in GFF format). The protein sequences are extracted from the annotation files, and the stop codons are removed. There was an average of 16,126 proteins per species.
- Step 2: **Predicted candidate effectors.** The effector prediction program EffectorP (version 2.0 and 3.0) [23, 24] was used to predict which proteins were effectors. This program identified 4,002 to 6,799 putative effector proteins in each of the six species (Table 3). There was an average of 4,869 proteins per species identified as candidate effectors.
- Step 3: **Predicted secretomes**. The secreted protein *in silico* identification program SecretSanta [25] is an efficient secretome prediction workflow that combines multiple command-line and web-interfaces tools such as SignalP [26], TargetP [27], TMHMM [28], TOPCONS [29] for signal peptide, motifs, and transmembrane domains prediction and WolfPsort [30] for the protein subcellular localization prediction. SecretSanta was used to identify which putative effectors are secreted from the fungus into the plant cell. The size of the predicted secretomes ranged from 308 to 463 proteins (Table 3). The ortholog identification program OrthoFinder [31] (with default parameters) was used to identify and analyze the distribution of putative secreted effectors for the six *Fusarium* species. The localization identification program LOCALIZER [32] identified potential subcellular localization signals on the putative secreted effectors that could direct them to the plant nuclei, chloroplasts, or mitochondria. There was an average of 384 proteins in a secretome per species.
- Step 4: **Predicted protein function**. The functional annotation program OmicsBox [33] predicted the functions of the putative effectors. OmicsBox utilizes CloudBlast software, which runs a Basic Local Alignment Search Tool (BLAST) analysis [34] against the NCBI-NR protein database. Putative functions were predicted by generating *Fusarium* secretome profiles for the six representative species and utilizing them as the primary, curated dataset for FPT. This dataset includes integrated profile list tables that provide detailed information and links to the FoldSeek and PanEffect tools for further analysis.

After the four-step process, the final set of predicted effectors ranged from 290 in *F. graminearum* to 462 in *F. oxysporum* (Table 3). These effectors are displayed in the ‘Effectors’ tab on the FPT.

#### Protein structure models

Protein structure models for the six representative species (*F. graminearum*, *F. fujikuroi*, *F. oxysporum*, *F. proliferatum*, *F. vanettenii*, and *F. verticillioides*) and four outgroups (*Arabidopsis thaliana*, *Homo sapiens*, *Saccharomyces cerevisiae*, *Schizosaccharomyces pombe*) were downloaded from the AlphaFold Protein Structure Database (https://alphafold.ebi.ac.uk/, Release 4). Additionally, ESMFold [35] was used to generate an independent set of 3D models for each proteome. For individual proteins, the 3D models derived from the two programs can differ because AlphaFold uses a deep learning approach that relies on multiple-sequence alignments, while ESMFold uses a protein language model. The use of the two independent sets of 3D models is expected to enhance FPT’s structural analysis capabilities. Supplementary Figure S2 provides a comparison of the average per residue confidence scores for the AlphaFold and ESMFold models for each of the six *Fusarium* proteomes. The software FoldSeek [10] facilitated the generation of both structural and sequence alignments. With FoldSeek, the 15,911 and 17,356 predicted protein structures for two agriculturally important species *F. graminearum* and *F. verticillioides*, respectively, were aligned to the predicted protein structures from the other four *Fusarium* species and four outgroup species. The resulting output was modified to highlight the top 25 hits (based on the highest alignment score) from each species, and to incorporate functional annotations from UniProt, species information, and a visual blue/red color gradient for intuitive interpretation of results [16, 36].

#### Pan-genome framework with variant effect scores

A diverse set of 22 *Fusarium* species, listed in Table 1, was selected to represent the *Fusarium* pan-genome. The selection criteria included high-quality genomes, identified as reference proteomes in UniProt, which provided a broad representation of the known sequence space for *Fusarium*. The phylogenetic relationships among these species are illustrated in Supplementary Figure S3. From this set, we selected a smaller group of six representative species: *F. graminearum*, *F. fujikuroi*, *F. oxysporum*, *F. proliferatum*, *F. vanettenii*, and *F. verticillioides*. This selection was based on capturing the presence and absence of gene space, particularly for mycotoxin biosynthetic gene clusters. Supplementary Figure S4 includes a presence-absence grid showing the distribution of selected mycotoxin biosynthetic gene clusters (Beauvericin/Enniatins, Fumonisins, Fusarins, Trichothecenes, and Zearalenone) across these six species. Supplementary Table S1 lists the members of the gene clusters, their presence or absence in these species, and the locus names which can be used as synonyms in the Fusarium Protein Toolkit (FPT). These six species were used in the effector annotation pipeline and served as protein target databases for FoldSeek.

The pan-genome was constructed using OrthoFinder [31] based on protein sequences obtained from UniProt [37]. This analysis resulted in 29,529 orthologous clusters (OrthoGroups), which constitute the pan-genome for this set of species and serve as a comprehensive framework for genetic diversity within the genus. Each OrthoGroup was subjected to multiple sequence alignments using FAMSA [38]. Finally, the variant effect scores were calculated using the Evolutionary Scale Modeling (ESM1b) protein language model [35] via the esm-variants tool. The scores predict the functional impact of amino acid substitutions. Scores above − 7 predict a benign effect of an amino acid substitution, whereas scores below − 7 predict a significant functional change. The variant effect scores were calculated for all 20 possible amino acid substitutions at each position in the two reference proteomes. This analysis encompassed over 127 million potential missense variants in the *F. graminearum* proteome and over 142 million in the *F. verticillioides* proteome, revealing the vast potential for genetic variation and its significant impact on phenotypic traits. These scores were cross-linked to the naturally occurring amino acid substitutions within the *Fusarium* pan-genome. FPT uses the PanEffect framework [39] to visualize and compare the variation to the reference proteomes, offering insights into the functional consequences of genetic diversity.

**Fig. 2:**
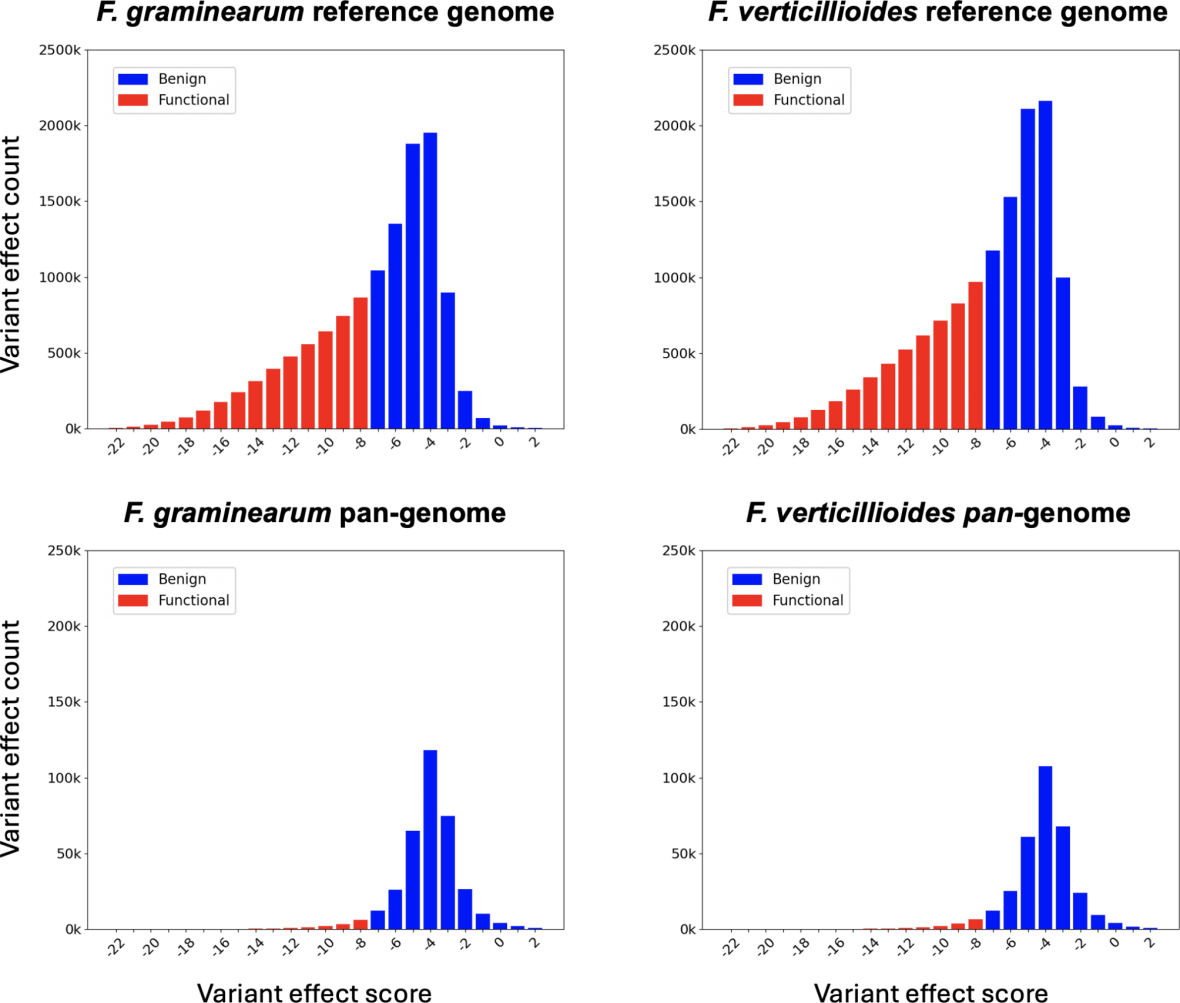
The distribution of variant effect scores in *Fusarium graminearum* and *Fusarium verticillioides*. The upper left panel shows the distribution of the variant effect scores for over 127 million missense variants among *F. graminearum* proteins. The upper right panel shows the distribution of the variant effect scores for over 142 million possible missense variants among *F. verticillioides* proteins. The bottom two panels illustrate the distribution of the variant effect scores of the actual missense variants between the reference proteins and the 22 proteomes in the *Fusarium* pan-genome. For each panel, the x-axis is labeled by the variant scores and the y-axis shows the count of variants with that score. Red bars have scores less than − 7 and are considered likely to have a functional effect. The blue bars have scores greater than or equal to -7 and are more likely to be benign

Notably, of the 127 million potential missense variants in *F. graminearum*, fewer than 28% (approximately 35.5 million) are observed within the pan-genome proteins, and for the 142 million possible variants in *F. verticillioides*, only 23% or 32.9 million are found in the pan-genome. See Fig. 2 for the distribution of the potential variant effect scores in *F. graminearum* and *F. verticillioides* alongside the distribution of naturally occurring variant effect scores in the *Fusarium* pan-genome. See Supplementary Figure S5 for the distribution of the potential variant effect scores for each of the 22 *Fusarium* proteomes. Beyond individual amino acid substitutions, our analysis of the variant effect scores across the *Fusarium* pan-genome offers an opportunity for broader discoveries about how genetic variations influence the biology of these species. For instance, the differential impact of these variants on protein function can indicate their roles in *Fusarium*’s adaptability to environmental stresses and host resistance mechanisms. Identifying patterns of detrimental effects among these variants could lead to breakthroughs in understanding pathogenicity mechanisms, offering new targets for disease control strategies.

### Utility and discussion

The Fusarium Protein Toolkit (FPT) interface, accessible at https://fusarium.maizegdb.org/, provides access to tools designed to facilitate the exploration of *Fusarium* protein structures and annotations. The interface is user-friendly, requiring no login, and offers underlying datasets for free download via a direct link on the webpage. The toolkit is organized into five main sections: Home, Effectors, FoldSeek, PanEffect, and Help. Each section is tailored to support different facets of *Fusarium* protein research effectively.

Figure 1, Panel B illustrates the FPT tools pipeline, which includes downloading protein sequences from UniProt, visualizing and searching sequences with FoldSeek, and assessing the functional impacts of missense variations. Additionally, Table 2 enumerates the tools and data availability for each *Fusarium* species represented within the FPT.

#### Home page overview

The FPT’s landing page is the home page, which provides quick access to the toolkit’s tools and features. Each section of the home page provides a concise overview of the toolkit’s capabilities with interactive components to search, download, or visualize the underlying data. The Home page incorporates direct search functionalities for the FoldSeek and PanEffect tools, allowing users to quickly query *Fusarium* gene or protein names. The table of annotated predicted effector genes is available through the menu or the “Fusarium Effector webpage” button on the homepage. The download section provides a bulleted list of downloadable datasets. The final two sections provide interactive widgets that display protein structures based on either the AlphaFold or ESMFold models.

#### Effectors page overview

The Effectors webpage provides a table of putative effector proteins for the six representative species of *Fusarium*: *F. graminearum*, *F. fujikuroi*, *F. oxysporum*, *F. proliferatum*, *F. vanettenii*, *F. verticillioides*. The tables are organized to show the confidence of the predictions, where the effector proteins are likely to be localized, functional annotations, and direct links to the other tools in the FPT. More specifically, each table includes the protein name, providing a primary reference identifier; linked UniProt IDs for in-depth protein information; apoplastic or intracellular localizations with the prediction probabilities and the amino acid positions of the domains, which are important for understanding effector modes of action; and additional localization details regarding the chloroplast, mitochondria, or nucleus to provide insight to the potential impacts on cellular processes. The table lists functional descriptions that give a concise overview of each protein’s potential functions and roles in *Fusarium*-host interactions. These descriptions are complemented by Gene Ontology (GO) [40] terms that give controlled vocabulary terms for biological processes, cellular components, and molecular functions. When applicable, enzyme codes are listed to indicate enzymatic activities. The final column in the table has direct links to FPT’s PanEffect and FoldSeek tools, alongside links to the AlphaFold Protein Structure Database. These links provide quick access to functional analysis and structural prediction which facilitate a streamlined and integrated workflow for researchers focused on plant pathology, mycology, and plant-microbe interactions.

By centralizing curated data of *Fusarium* effector proteins, the Effectors webpage enables users to quickly access detailed information, compare effector functions and localizations, and explore links to external databases for extended research. The integration of direct links to analytical tools and databases provides a transition from gene identification to functional and structural analysis, which is important to advance our understanding of *Fusarium* effector proteins and their roles in plant disease.

#### FoldSeek (protein structure search) overview

**Fig. 3:**
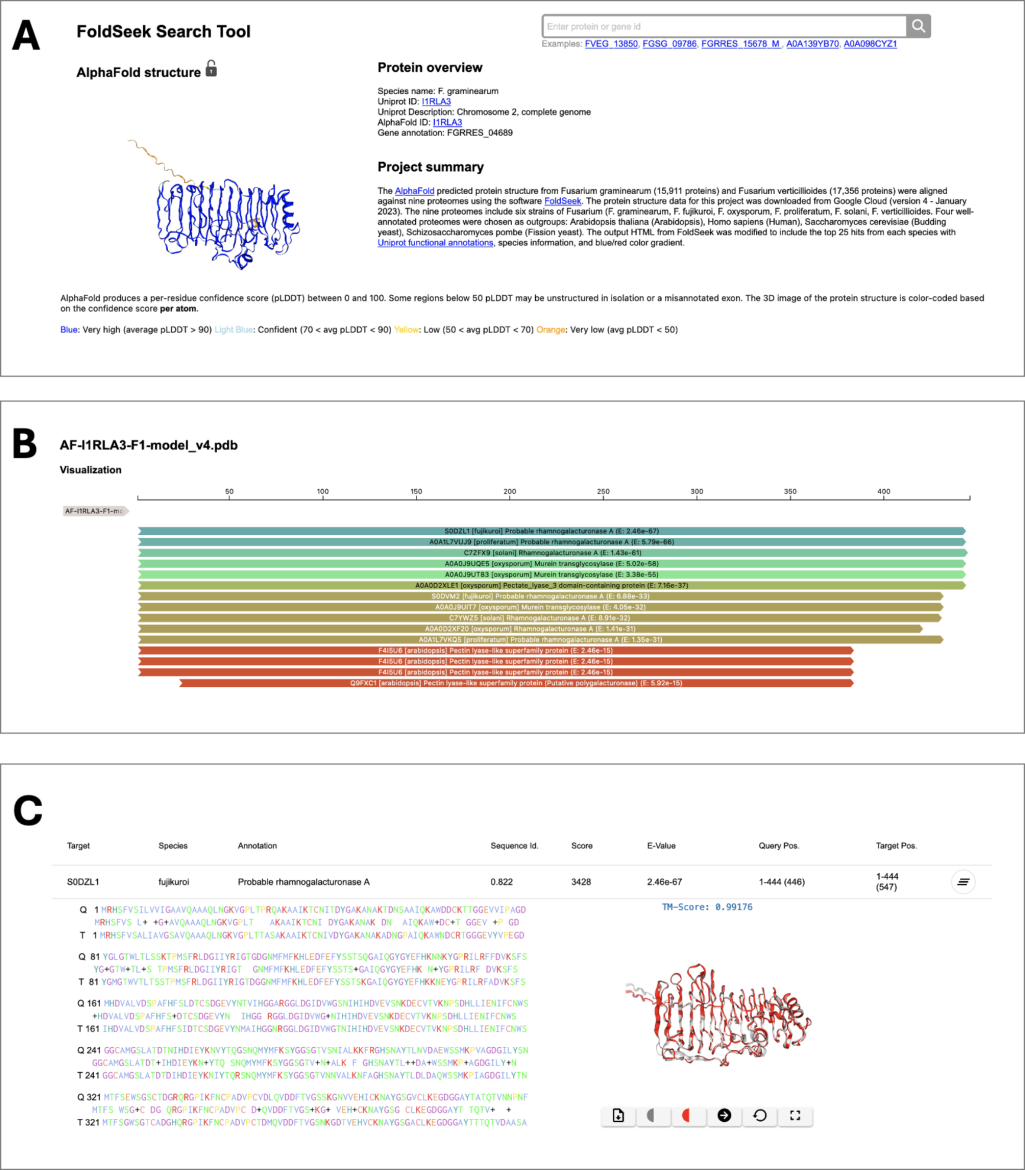
Example page of *Fusarium* FoldSeek search tool for the *F. graminearum* gene FGRRES_04689 (UniProt: I1RLA3). (A) The protein overview section on the top of the page, which includes the Project summary, Protein overview, and AlphaFold structure. (B) A zoomed-in view of the scores for the 15 top hits of UniProt proteins aligned to FGRRES_04689; the top hit is the S0DZL1 protein from the *F. fujikuroi* annotated as a “Probable rhamnogalacturonase A.” Clicking on the panel containing the alignment score for takes the user to the protein sequence alignment. (C) The structural superposition of the top hit of the *F. graminearum* and *F. fujikuroi* proteins. The table provides additional metrics on the alignment and an interactive structure view

The FoldSeek Search Tool is designed to provide insights into protein structures and sequence alignments (Fig. 3). Utilizing the FoldSeek software, this resource enables the comparison of AlphaFold protein structure alignments from either *F. graminearum* or *F. verticillioides* with nine proteomes. This selection of proteomes includes six *Fusarium* species and four outgroup proteomes from species such as *A. thaliana* and *H. sapiens*, facilitating a broad comparative analysis. The tool offers visualizations of protein structure alignments, including 3D views of *Fusarium* protein structures predicted by AlphaFold, to help in understanding protein configurations. It also provides access to detailed protein information, such as UniProt IDs, gene names, Pfam domains, and functional annotations. The tool presents structural alignment quality in a color-coded format, making it easier to identify the level of similarities and differences between orthologous proteins. An interactive feature allows users to click on these color-coded bars to access more detailed comparisons of sequences and the superposition of the structural alignments between different species.

The FoldSeek Search Tool aids researchers by providing a platform to visualize, analyze, and compare protein structure alignments. This tool will be particularly useful for comparative studies of fungi by identifying orthologs, inferring function, exploring structural domains, and comprehending the evolutionary relationships among proteins that were not evident through sequence alignments alone.

#### PanEffect (variant effect viewer) overview

**Fig. 4:**
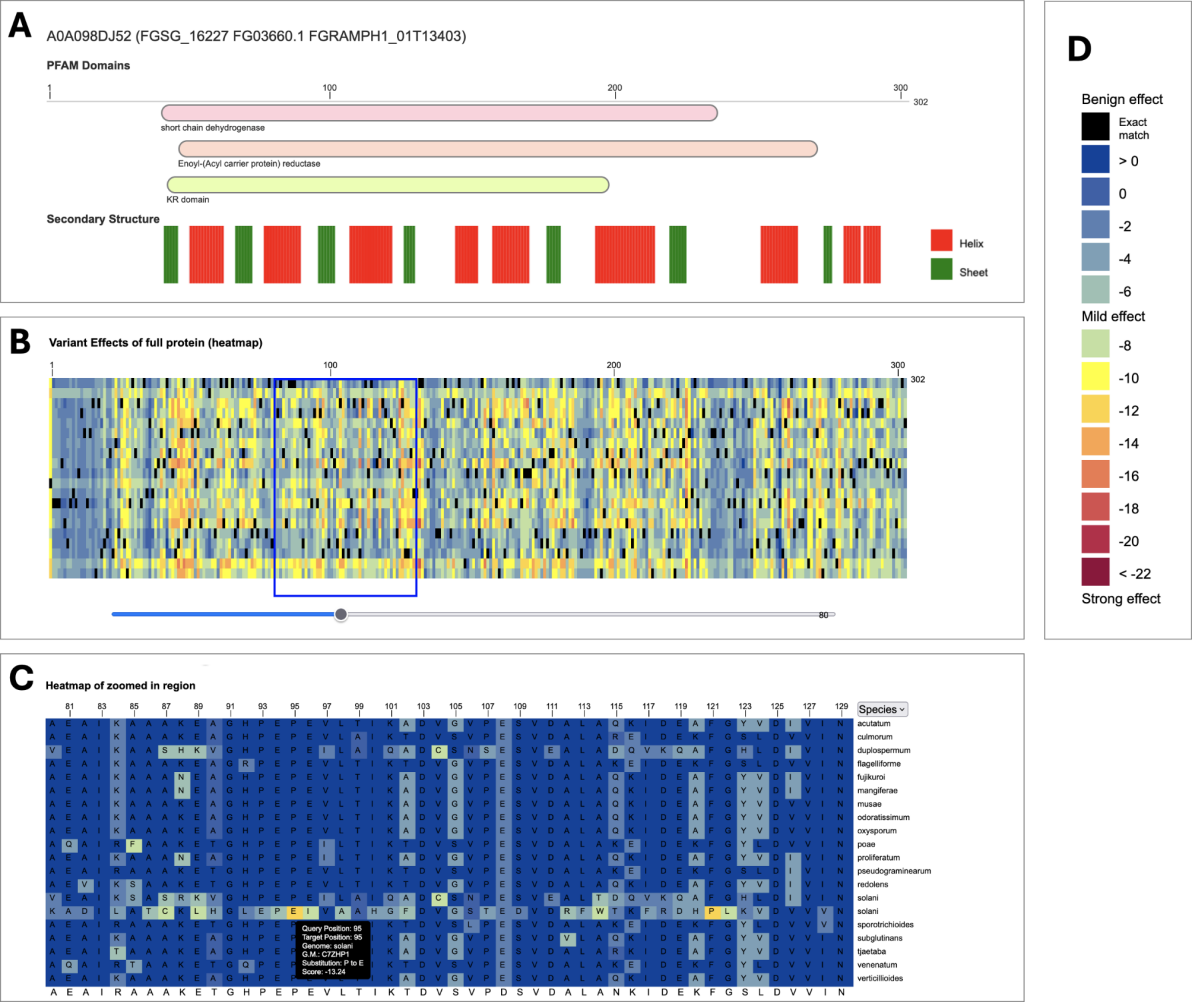
Example page of *Fusarium* PanEffect tool for the *F. graminearum* gene FGSG_16227 (UniProt: A0A098DJ52). The PanEffect tool has two major views showing the heatmaps of variant effect consequence scores at the reference genome level and pan-genome level. (**A**) A snapshot of the Pfam domains and predicted secondary structures for the given protein. This view is available on each page to give some functional and structural context to the coding variants. (**B**) A snapshot of the zoomed-out heatmap view of the “Variants effects within a genome” tab. It displays a heatmap of all possible coding variants color-coded based on how likely it will affect the protein’s function. (**C**) A snapshot of the zoomed-in heatmap view of the “Variants effects within gene families” tab. It shows a heatmap of the naturally occurring variants found in a gene family across 22 diverse *Fusarium* genomes, where variants that are the same as the reference genome (in this case *F. graminearum)* are shown in dark blue. All other variants are color-coded based on how likely it will affect the protein’s function. Mousing over a position on the heatmap shows additional details including the amino acid substitution and predicted score. (D) The legend of the variant effect scores where scores above − 7 indicate benign outcomes, while scores below − 7 suggest possible phenotypic effects

The PanEffect tool (Fig. 4) is a resource designed for in-depth analysis of the potential impacts of missense variants on *Fusarium* proteins. It provides users with an interactive interface comprising four specialized views. The Search function enables users to make queries based on gene names and protein identifiers. The Gene Summary section presents an overview of the gene and protein annotations. In the Variant effects within a genome view, detailed heatmaps show the predicted functional impact of all possible amino acid substitutions for *F. verticillioides* or *F. graminearum* proteins. These heatmaps, which transition from blue to red to indicate impact severity, provide insights into each position of the reference protein and the possible amino acid substitutions. Additionally, the tool allows for the exploration of Variant Effects across the pan-genome with additional heatmaps that reflect the effects of natural variants on *Fusarium* proteins across orthology-based gene families built on 22 *Fusarium* species. In addition, over 3 billion effect scores for nearly 330,000 proteins across the 22 species are available as downloads.

The PanEffect tool leverages protein language models to provide a nuanced understanding of how missense variants affect *Fusarium* proteins. These insights can empower researchers to predict and interpret the functional consequences of genetic variants. Moreover, the tool can support a wide range of comparative studies and help make predictions on how variation affects proteins involved in *Fusarium*-plant interactions. Descriptions of the tools, data sources, and available downloads used in PanEffect can be found in the Help tab.

#### Help page overview

The help section offers summaries of all the FPT components and includes detailed descriptions and links to the data sources, tools, downloads, and references used to develop FPT. The Help page also includes a table of *Fusarium* species used to develop this resource with links to NCBI and UniProt.

#### Comparison to existing databases

The Fusarium Protein Toolkit (FPT) provides a unique and integrated platform for researchers focusing on *Fusarium* species, distinguishing itself from other existing databases and resources. Other resources offer specific functionalities, but FPT’s specialization makes it particularly valuable for *Fusarium* research. Here are some resources and databases that offer similar or complementary functionality. FungiDB (https://fungidb.org/) [41] is a resource that offers genomic data, gene annotations, nomenclature, a genome browser, and BLAST tools for a wide range of fungal species. It provides a foundational understanding of genomic elements but lacks the protein-specific tools offered by FPT. UniProt (https://www.uniprot.org/) is a widely-used repository that provides detailed proteomics data, including protein sequences, structures, functional information, and various annotations. While it offers extensive data, it does not specifically cater to *Fusarium* proteomes nor does it integrate the unique combination of structural and functional analyses available in FPT. The AlphaFold Protein Structure Database (https://alphafold.ebi.ac.uk/) allows for the visualization and download of protein structures. It is now integrated with AlphaMissense, which categorizes potential protein variations as either ‘likely pathogenic’, ‘likely benign’, or ‘uncertain’, providing a score to estimate the likelihood that a variant is pathogenic. While these tools are valuable for structural biology, they do not provide comprehensive proteomics-level data specifically for *Fusarium* species. Notably, AlphaMissense predictions are currently available only for human proteins. Additionally, the absence of integration with other *Fusarium*-specific annotations restricts its utility for targeted research in this area. FoldSeek (https://search.foldseek.com/search) excels in rapidly comparing protein structures across known protein sequences in biological databases. Despite its efficiency, it is not tailored to integrate *Fusarium-*specific annotations and lacks the detailed functional analyses that FPT provides, making it less suitable for focused studies on *Fusarium* proteins. PredictProtein (https://predictprotein.org/) [42] and the Ensembl Variant Effect Predictor (VEP) [43] are two examples that use AI-driven methodologies to provide functional and structural annotations for a wide range of proteins, including heatmaps that show the functional effects of point mutations. While they are similar to the PanEffect feature in FPT, these resources are general-purpose tools not specifically focused on *Fusarium*. Additionally, there analyses are not all precomputed, which can slow down the workflow compared to the precomputed datasets available in FPT.

FPT is uniquely designed for *Fusarium* proteomes, offering a tightly integrated platform that combines functional annotations, 3D structure visualization and search, pan-genome analysis, and the assessment of functional effects of variations across different *Fusarium* species. The datasets are curated and precomputed, allowing for rapid and efficient analysis. This specialized focus and integration make FPT a valuable resource for researchers dedicated to understanding *Fusarium* proteins and their roles in plant disease interactions.

#### Case Study: Using the Fusarium Protein Toolkit to Analyze the FVEG_03351 gene

**Fig. 5:**
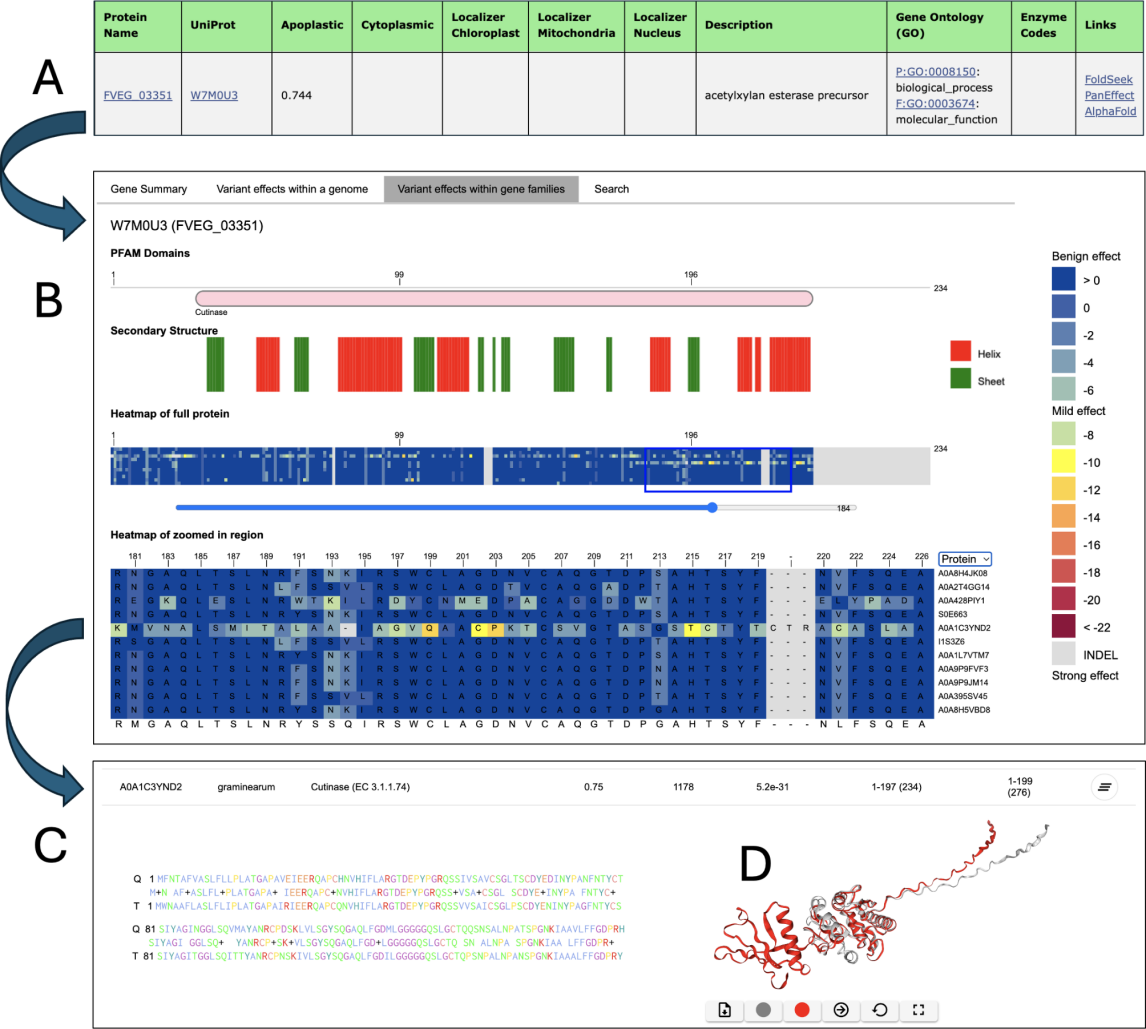
Using the Fusarium Protein Toolkit to Analyze FVEG_03351. The figure shows how the Fusarium Protein Toolkit can be used for the analysis of the FVEG_03351 gene (UniProt: W7M0U3) from *F. verticillioides*. FVEG_03351 is annotated as a cutinase protein, a type of inducible extracellular enzyme secreted by microorganisms that can degrade plant cell walls. (A) The Effector annotation pipeline identifies FVEG_03351 as an effector gene. (B) The PanEffect tool for FVEG_03351 reveals the presence of the cutinase domain and predicted secondary structures. The tool also displays color-coded variant effect consequence scores in heat maps, including the “Variant effects in the gene family” tab, which shows the consequence scores of missense variants across 11 members of the gene family that were calculated across 22 *Fusarium* proteomes. In this example, the protein A0A1C3YND2 from *F. graminearum* contains several variants predicted to have a strong functional effect (zoomed-in region). (C) A sample output of the FoldSeek tool for proteins W7M0U3 (query protein) and A0A1C3YND2 (target protein) which is also accessible through the Links column in the Effectors table. D). In the structure superposition view, the query protein W7M0U3 is shown in gray, and A0A1C3YND2 in red. The structure shows the portion of A0A1C3YND2 from position 190 to the terminal of the protein conforms to a different structure as W7M0U3

A key application of FPT is to visualize coding changes and correlate them with both predicted functional consequences and structural differences. Figure 5 presents the analysis of the gene FVEG_03351 (UniProt: W7M0U3) from *F. verticillioides*, utilizing the capabilities of the Fusarium Protein Toolkit. FVEG_03351 is annotated as a cutinase protein [44], which is an inducible extracellular enzyme secreted by microorganisms capable of degrading plant cell walls. This characteristic underscores its potential role in host-pathogen interactions, particularly in agricultural settings where *Fusarium* species are known pathogens. The toolkit’s Effector Annotation Pipeline categorizes FVEG_03351 as an effector gene (Fig. 5A), suggesting its involvement in pathogenicity by facilitating the degradation of plant host tissues, thereby promoting infection. Utilizing the PanEffect tool (Fig. 5B), the cutinase domain and its predicted secondary structures within FVEG_03351 are shown. This tool also displays heat maps that are color-coded to represent variant effect consequence scores. In the “Variant Effects in the Gene Family” tab, these scores reveal the impact of missense variants across gene family members of other *Fusarium* species. The protein A0A1C3YND2 from *F. graminearum* has a series of variants from position 180 to the end of the protein (shown in the zoomed-in heatmap) that are predicted to strongly affect its function.

The FoldSeek tool (Fig. 5C) can be used to examine the structural alignments and identify structural changes caused by the coding differences between the query protein W7M0U3 in *F. verticillioides* and the target protein A0A1C3YND2 in *F. graminearum*. This analysis is valuable for understanding the conformational variants that might influence protein function and interaction dynamics. The structural superposition view in the FoldSeek tool (Fig. 5D) is depicted with the query protein W7M0U3 in gray and A0A1C3YND2 in red. This comparison visually emphasizes the structural differences between the two proteins, specifically the stretch from position 180 to the terminal end of A0A1C3YND2 which overlaps the region found in PanEffect. The structural differences observed are substantial for this region and provide further support for the possibility of functional or phenotypic differences between the two proteins. This analysis used the FPT to identify structural and functional differences of an important gene implicated in plant disease. By detailing the contributions of each component of the toolkit, this figure underscores the multifaceted approach required to understand the roles of such proteins in pathogenicity, offering insights that are crucial for developing targeted strategies against *Fusarium*-related plant diseases.

## Conclusions

The Fusarium Protein Toolkit has the potential to be a valuable resource in fungal pathogen research. It offers a comprehensive suite of tools to explore protein structures, variant effects, and annotated effector proteins of *Fusarium*. Researchers can visualize and analyze protein structures from AlphaFold and ESMFold models, comparing them across diverse *Fusarium* species and outgroups. The toolkit also leverages protein language models to predict the functional impact of missense variants, which has the potential to provide valuable insights into how variants could affect protein function. Additionally, the PanEffect tool allows researchers to explore the effects of natural variations within a pan-genome based on 22 *Fusarium* species, which could aid the understanding of the impact of genetic diversity on the biology of these fungi.

By offering these capabilities, FPT has the potential to deepen our understanding of the pathogenic mechanisms of *Fusarium*, which could in turn facilitate the identification of proteins that can be used as targets to control *Fusarium*-incited crop diseases and mycotoxin contamination and thereby ensure food security in a changing climate. FPT takes a step forward not only by offering these *Fusarium*-specific insights but also by being built upon the same frameworks and tools used for the model host plant species, maize. This shared infrastructure lays the groundwork for future work aimed at understanding host-pathogen interactions, by enabling direct comparisons of protein structures and functionalities between *Fusarium* and maize, researchers can gain a deeper understanding of how these organisms interact at the molecular level. Knowledge of *Fusarium*-maize interactions has the potential to identify vulnerabilities in these interactions, which are potential targets for the development of control strategies.

## Electronic supplementary material

Below is the link to the electronic supplementary material.

Supplementary Material 1

Supplementary Material 2

## Data Availability

The Fusarium Protein Toolkit is freely accessible at https://fusarium.maizegdb.org/ and is maintained by MaizeGDB. The PanEffect framework is available at https://github.com/Maize-Genetics-and-Genomics-Database/PanEffect. The underlying data generated from the artificial intelligence and bioinformatics approaches are found in the *Fusarium* data folder in the Artificial Intelligence section of the MaizeGDB download page (https://maizegdb.org/download*).*
