# Supplementary material for: ​Fusarium Protein Toolkit: a web-based resource for structural and variant analysis of Fusarium species
Source: BMC Microbiol. 2024 Sep 6;24:326. doi: 10.1186/s12866-024-03480-5 (PMC11378500; doi:10.1186/s12866-024-03480-5)
Supplement: Supplementary file 1 — Supplementary Material 1 [file 12866_2024_3480_MOESM1_ESM.pdf]

Dataset	Method	Format	FPT curated
Sequences	Download	FASTA	✖
3D structures	AlphaFold	PDB	✖
3D structures	ESMFold	PDB	☑
Variant Effects	esm-variant	CSV	☑
Orthology groups	OrthoFinder	TXT	☑
Effectors	EffectorP	TSV	☑
Secretomes	SecretSanta	TSV	☑
Functions	OmicsBox	TSV	☑

Supplementary Figure S1: Overview of the Fusarium Protein Toolkit (FPT) Datasets. This table shows the datasets incorporated into the Fusarium Protein Toolkit. It outlines each dataset's type, the method utilized for its generation, the format of the data, and whether the dataset was curated specifically for this project (FPT curated). Notably, the protein sequences and AlphaFold structures were not generated within this project but were obtained externally; sequences were downloaded directly from GenBank or UniProt, and AlphaFold structures were downloaded from the AlphaFold Protein Structure Database.

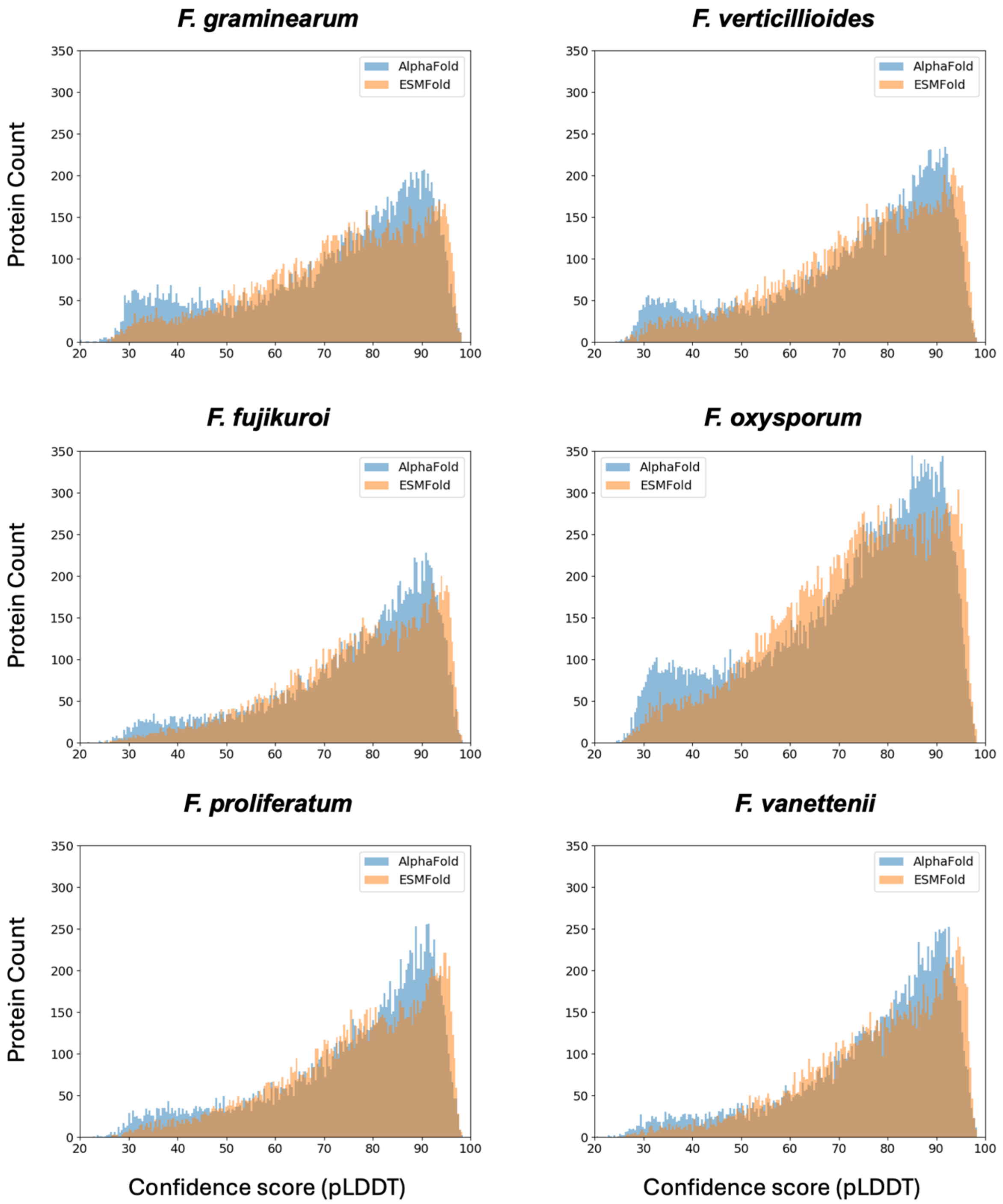

Supplementary Figure S2: Comparison of the distribution of confidence scores from AlphaFold and ESMFold. The Fusarium Protein Toolkit provides both AlphaFold and ESMFold structures. AlphaFold is a deep learning system approach that relies on multiple-sequence alignments while ESMFold uses language models. Individual plots show the distribution of average residue confidence scores (PLDDT) for each of the six representative *Fusarium* species. AlphaFold predicted a greater number of proteins with a high confidence score (≥ 70 pLDDT) but takes up to 60 times longer to make each prediction.

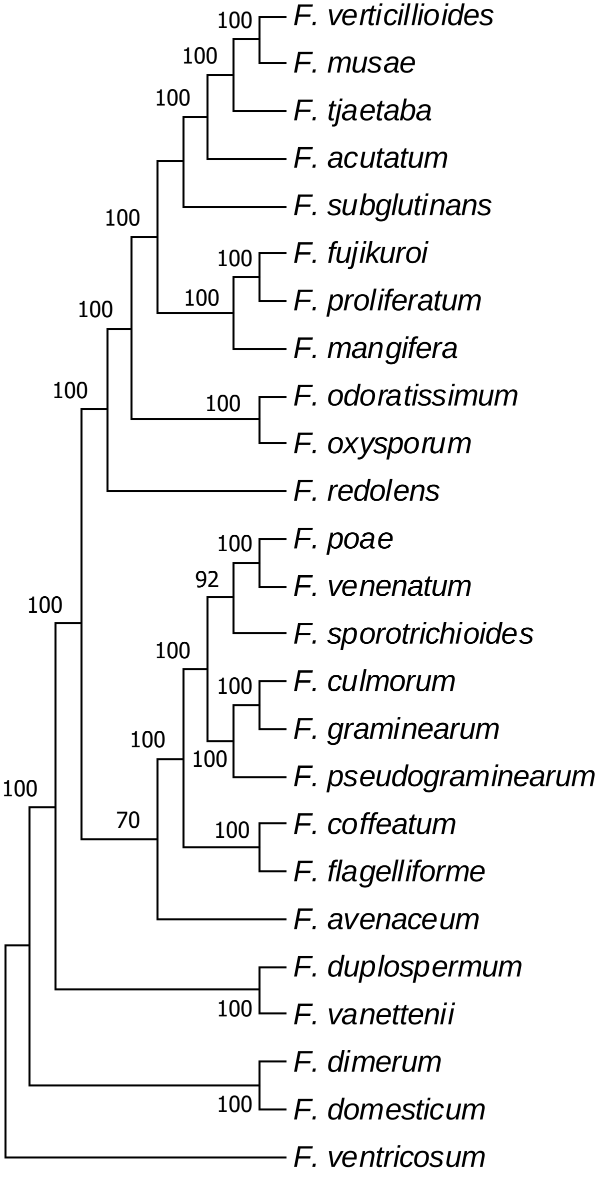

Supplementary Figure S3: Phylogenetic tree of the 22 *Fusarium* species examined for the Fusarium Toolkit. The tree was inferred using concatenated alignments of DNA sequences of exon sequences of the 19 genes employed by Geiser et al. (2021) The three species at the base of the tree (*F. dimerum*, *F. domesticum* and *F. ventricosum*) were included as outgroups in the phylogenetic analysis. The analysis was done using IQ-Tree as outlined by Geiser et al. (2021).

Reference

Geiser DM, Al-Hatmi A, Aoki T, Arie T, Balmas V, Barnes I, Bergstrom GC, Bhattacharyya MKK, Blomquist CL, Bowden R et al. 2021. Phylogenomic analysis of a 55.1 kb 19-gene dataset resolves a monophyletic *Fusarium* that includes the *Fusarium solani* Species Complex. Phytopathology 111:1064-1079. 10.1094/phyto-08-20-0330-le

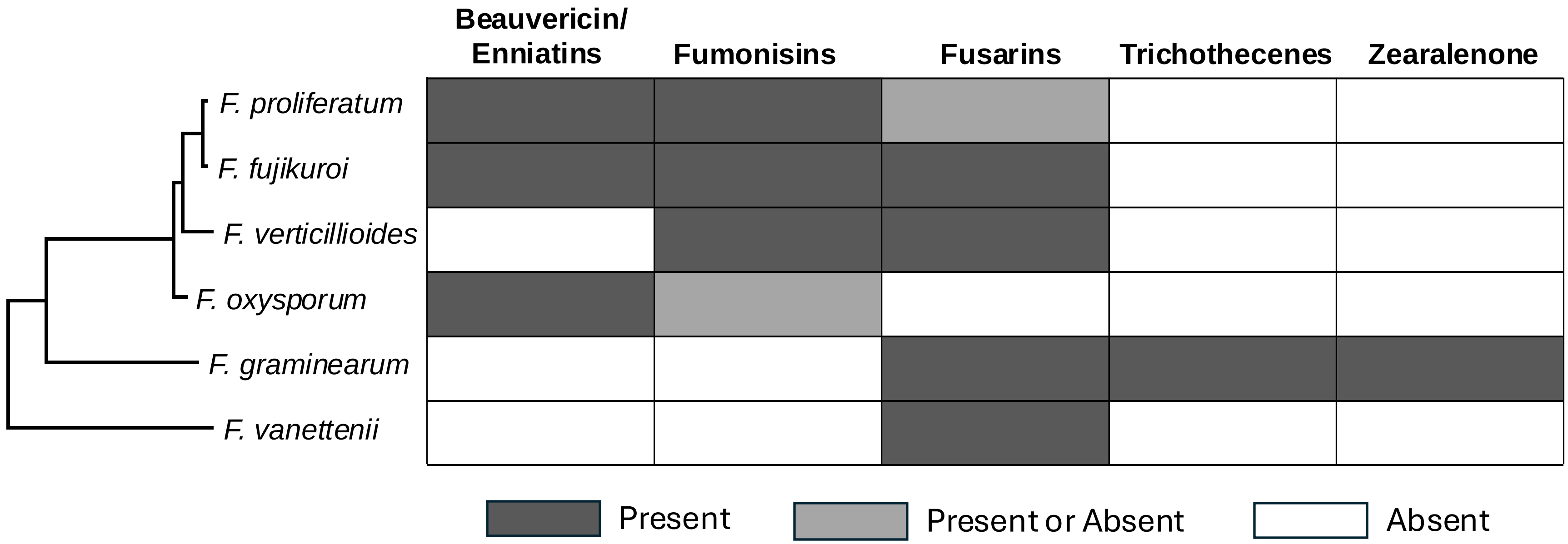

Supplementary Figure S4: Presence-absence grid showing the distribution of selected mycotoxin biosynthetic gene clusters in the six *Fusarium* species used in the effector annotation pipeline. The gene clusters were detected by AntiSmash analysis and confirmed by manual BLAST analysis as previously described (Kim et al. 2020). In the key below the presence -absence grid, “Present or Absent” indicates that a functional cluster was detected in one or more isolates examined but not detected in one or more other isolates. The tree to the left of the grid was inferred using the 19-gene dataset as described by Geiser et al. (2021).

References

Geiser DM, Al-Hatmi A, Aoki T, Arie T, Balmas V, Barnes I, Bergstrom GC, Bhattacharyya MKK, Blomquist CL, Bowden R et al. 2021. Phylogenomic analysis of a 55.1 kb 19-gene dataset resolves a monophyletic *Fusarium* that includes the *Fusarium solani* Species Complex. Phytopathology 111:1064-1079. 10.1094/phyto-08-20-0330-le

Kim HS, Lohmar JM, Busman M, Brown DW, Naumann TA, Divon HH, Lysoe E, Uhlig S, Proctor RH. 2020. Identification and distribution of gene clusters required for synthesis of sphingolipid metabolism inhibitors in diverse species of the filamentous fungus Fusarium. BMC Genomics 21:510. 10.1186/s12864-020-06896-1

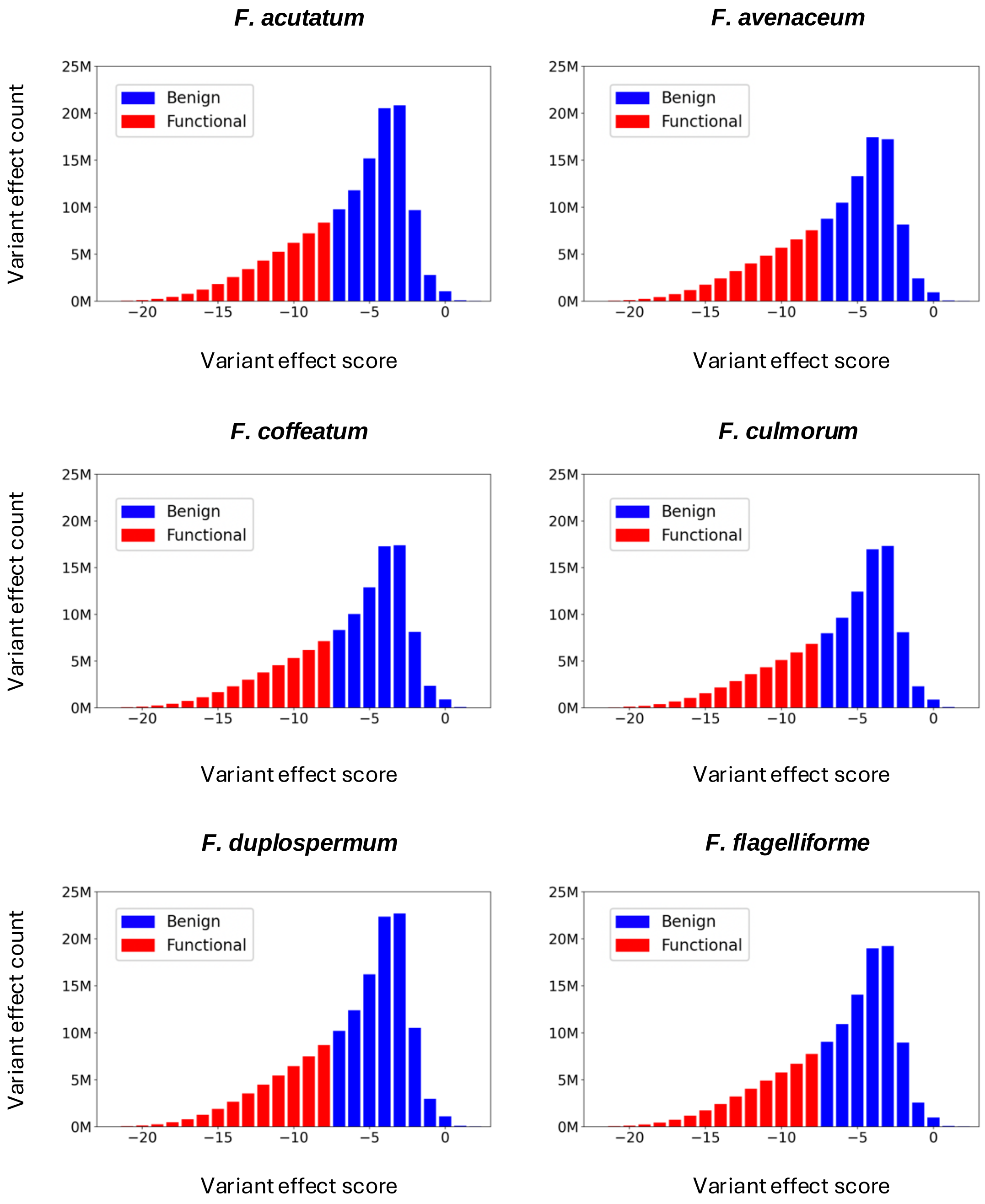
F. acutatum
F. avenaceum
F. coffeatum
F. culmorum
F. duplospermum
F. flagelliforme
Benign
Functional
Variant effect count
Variant effect score
25M
20M
15M
10M
5M
0M
−20
−15
−10
−5
0

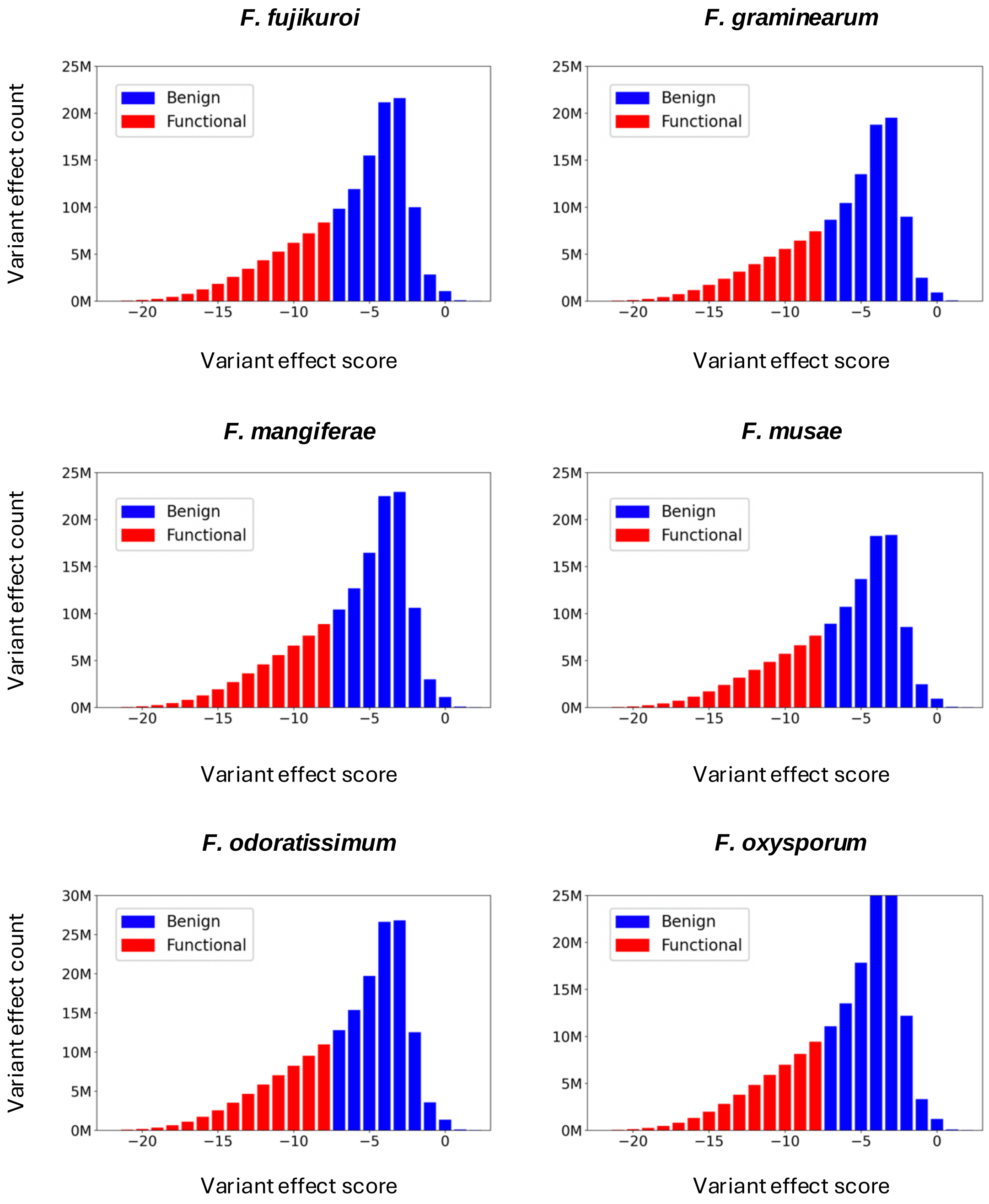
F. fujikuroi
F. graminearum
F. mangiferae
F. musae
F. odoratissimum
F. oxysporum
Benign
Functional
Variant effect count
Variant effect score
0M
5M
10M
15M
20M
25M
30M
−20
−15
−10
−5
0

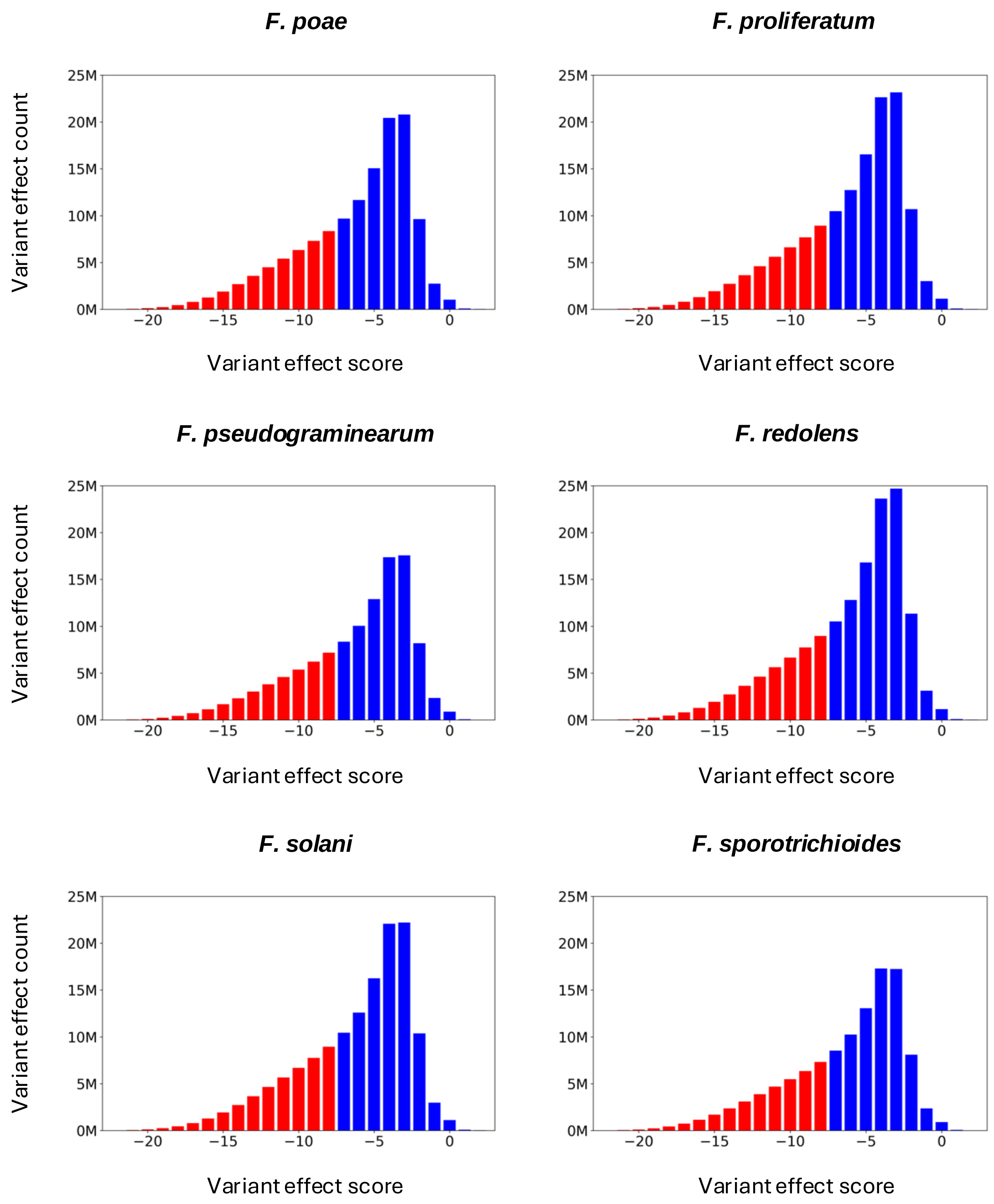
F. poae
F. proliferatum
F. pseudograminearum
F. redolens
F. solani
F. sporotrichioides
Variant effect count
Variant effect score
25M
20M
15M
10M
5M
0M
−20
−15
−10
−5
0

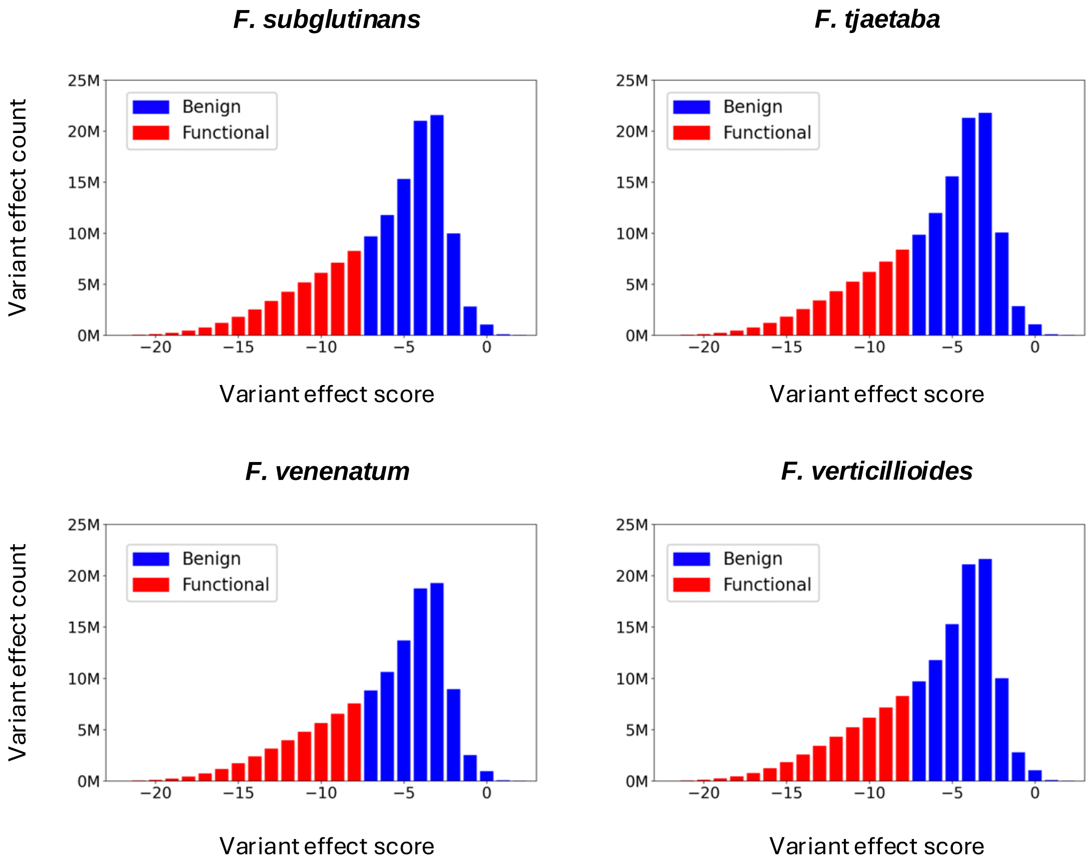

Supplementary Figure S5: The distribution of variant effect scores across the 22 Fusarium species. Each panel shows the distribution of the variant effect scores for every possible missense variant among for the 22 Fusarium species. For each panel, the x-axis is labeled by the variant scores and the y-axis shows the count of variants with that score. Red bars have scores less than -7 and are considered likely to have a functional effect. The blue bars have scores greater than or equal to -7 and are more likely to be benign.
